# Commentary: Sweet taste is complex: Signaling cascades and circuits involved in sweet sensation

**DOI:** 10.3389/fnhum.2023.1167749

**Published:** 2023-03-29

**Authors:** Daniel M. Ennis

**Affiliations:** The Institute for Perception, Richmond, VA, United States

**Keywords:** sweet, taste, receptor, transducer, pharmacokinetics, chemosensory psychophysics, models, agonist

## Introduction

In their otherwise comprehensive review of signaling mechanisms in sweet taste, von Molitor et al. (2021) missed research on human sweet taste modeling that originated in pharmacokinetics. This research predates the receptor characterization that they reviewed by about a decade and was conducted in humans. The von Molitor et al. paper, published in a journal devoted to human neuroscience, appropriately begins with a background on sugar consumption and health in humans. The paper goes on to provide a brief overview of the severe limitations associated with human experimentation and concludes that “taste-related signaling mechanisms have been studied mainly in rodents, although there are major species-related differences”. This commentary will provide a brief account of the methodology, models, and conclusions from the omitted research to augment the von Molitor et al. review.

## Transduction mechanisms in human taste

Black was awarded the Nobel prize in Physiology or Medicine for the science behind the two blockbuster drugs of the twentieth century: propranolol (a beta blocker) and cimetidine (a histamine receptor antagonist). His Nobel lecture (Black, 1989), entitled *Drugs from emasculated hormones: the principle of syntopic antagonism*, vividly entertained the idea of a two-stage process for creating blockers. This process consisted in fruitful receptor binding without subsequent transducer action, and thus separated and exploited these two stages. Black and Leff (1983) had previously published a mathematical model that provided parameters for the two stages called *affinity* and *efficacy* and these refer to the initial receptor binding and the subsequent participation of a transducer, such as a G-protein. The possible common evolutionary ancestry of the mechanisms for heart stimulation, vision, taste, and smell (Lancet et al., 1988) suggested that this two-stage model may have value in modeling the chemical senses, including sweet taste (Pace et al., 1985; Anholt, 1987). Support for this hypothesis had not yet been demonstrated, however, in humans.

## Pharmacokinetic models for sweet taste mixtures in humans

**Figure 1:**
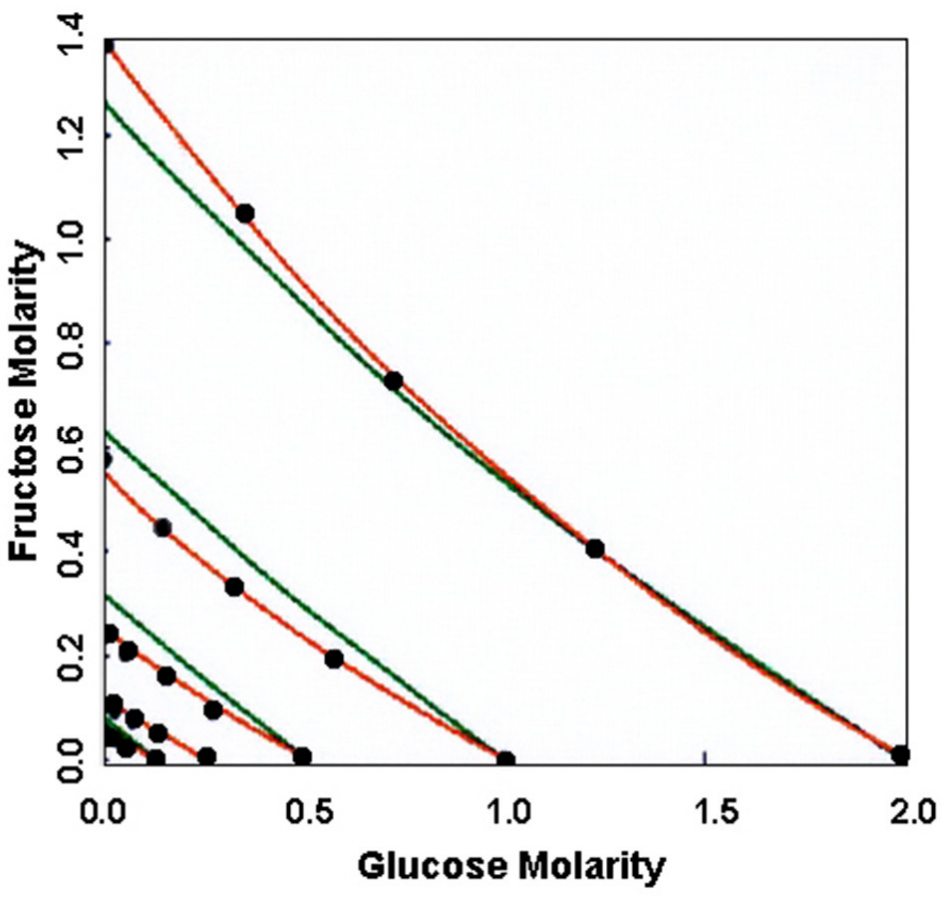
Plots of the concentrations of glucose and fructose in mixtures that are equally sweet to target concentrations of glucose alone (the black dots). Also shown are the fits of the models given in Table 1 for the independent receptor model (shown in green) and the independent receptor-transducer model (shown in red). The data were obtained from DeGraaf and Frijters (1986) and the fit of the models from Ennis (1991).

Developing and testing a model for smell and taste in humans poses some challenges because human experimentation necessarily involves chemosensory psychophysics. This means that measurements are made at some distance from the site of action. An opportunity to expand and test an extension of Black and Leff's model for single substances to mixtures of agonists occurred when DeGraaf and Frijters (1986) published experimental results on the points of subjective equality of glucose and fructose mixtures following Beidler (1962). These authors considered common receptor binding without the participation of a transducer and did not develop and test models with independent receptors or models with common or independent receptor-transducer systems. Common receptor binding means that competitive agonism occurs between the two substances and independent binding means that it does not. Independent receptor-transducer systems are those in which competition does not occur between the mixture components for the receptor or transducer. Plots of equal sweetness of the single substances and mixtures of them are called isoboles (Ennis, 1991), which may be linear (no synergy) or non-linear (synergy). An advantage of comparative mixture experiments is that it is not necessary to know the precise function that connects the periphery to the percept, only that it is monotonic. The modeling challenge was to develop equations from the law of mass action that included the binding constants for each substance to independent or common receptors and for independent or common receptor-transducer systems, if they occurred. The equations for the four derived models are given in Table 1 (summarized from Ennis, 1991, 1996). Figure 1 shows the fit of the data to independent receptors and independent receptors coupled to a transducer (Ennis, 1991, Figure 2). The common receptor and common receptor-transducer models (involving competitive agonism) were ruled out because they cannot explain synergy since their isoboles are linear. The best-fitting model was the independent (non-competitive) receptor-transducer model which was significantly superior to the independent receptor model without participation of a transducer as shown in Figure 1. Other models, such cooperative binding models where both substances may act together on receptor sites, were less parsimonious than the simple binding, independent receptor-transducer model. This research showed, for the first time, that human chemical sensing involves a transducer entity, such as a G-protein. Model parameters for affinity and efficacy in humans, as defined by Black and Leff (1983), were determined by Ennis (1991, 1996) using non-linear least squares to determine the model parameters for each model using the Levenberg-Marquart algorithm. It was shown that fructose is sweeter than glucose because its receptor affinity is higher than glucose, although its efficacy or transducer engagement, is weaker. Subsequently, the mixture models were used to study synergy between fructose, sucralose and high potency sweeteners (Wolf et al., 2010).

**Table 1:** Receptor and receptor-transducer models assuming common and independent systems.

	**Common**	**Independent**
Receptor	[*B_*m*_*] = *k* ([*A*] – [*A_*m*_*]), *k* = $\frac{K_{a}}{K_{b}}$	[*B_*m*_*] = $\frac{k\;\left(\left[A\right]\;-\;\left[A_{m}\right]\right)}{1+2K_{a}\left[A_{m}\right]+K_{a}^{2}\left[A\right]\left[A_{m}\right]}$
Receptor-transducer	[*B_*m*_*] = $\left[\frac{\alpha \;\left(\left[A\right]\;-\;\left[A_{m}\right]\right)}{1+\beta \left[A\right]}\;\right]$, where β= *K_*a*_ – αK_*b*_ α =* $\frac{K_{a}K_{ar}}{K_{b}K_{br}}$	[*B_*m*_*] $=\frac{\alpha \;\left(\left[A\right]\;-\;\left[A_{m}\right]\right)}{\theta }$ θ = 1 + β[*A*]+[*A*_*m*_]{*K*_*a*_(1+2τ)+α*K*_*b*_}+ [*A*]$\left[A_{m}\right]\left\{K_{a}^{2}+{\left(1+\tau \right)}^{2}\right\}$*α =* $\frac{K_{a}K_{ar}}{K_{b}K_{br}},$ β= *K_*a*_ – α*K*_*b*_*, and τ = *K*_*ar*_[*R*]

[*A*_*m*_] and [*B*_*m*_] refer to the concentrations of glucose and fructose in mixtures, respectively. [A] refers to a target concentration of one of the mixture components (glucose) and is a known constant to which the mixtures are compared. *K*_*a*_ and *K*_*b*_ refer to binding constant to receptors (affinity). *K*_*ar*_ and *K*_*br*_ refer to agonist-receptor binding to a transducer (efficacy). *R* refers to the concentrations of the receptors in the independent systems which are assumed to be equal (summarized from Ennis, 1991, 1996).

## Conclusions

von Molitor et al. limited their consideration of human psychophysical tests to detection, intensity and hedonic measurements from subject reports. However, a much richer vein of exploration in humans can be pursued by considering how to apply models developed in pharmacokinetics and expanded to include mixtures of substances with similar effects, such as sweeteners or drugs. From this research, it was shown that a transducer exists in human sweet taste, that fructose and glucose operate on independent systems which leads to synergy, and that the affinity of fructose is greater, and its efficacy weaker, than glucose. This research put chemosensory psychophysics on a molecular foundation.

## Author contributions

The author confirms being the sole contributor of this work and has approved it for publication.
